# Ultrasound-triggered tumor therapy with doxorubicin-liposome-microbubble complexes in a subcutaneous murine colon adenocarcinoma model

**DOI:** 10.1186/2050-5736-3-S1-P66

**Published:** 2015-06-30

**Authors:** Alexander Klibanov, Zhongmin Du, Galina Diakova

**Affiliations:** 1 University of Virginia, Charlottesville, Virginia, United States

## Background/introduction

Triggered release of drugs from carrier systems *in vivo* is widely investigated for targeted tumor therapy. During the past several years, an ultrasound-sensitive liposome-microbubble pendant carrier has been developed: microbubbles, decorated with liposomes that carry e.g., doxorubicin, release the drug in response to ultrasound pulse.

However, due to low drug load, those particles were not successful for tumor therapy *in vivo*. In this study, we are able to prepare particles with high doxorubicin load, and achieve successful inhibition of tumor growth in a subcutaneous murine tumor model.

## Methods

Decafluorobutane microbubbles were stabilized with a shell consisting of DSPC, PEG stearate and biotin-PEG-DSPE (20:20:1 mass ratio). Liposomes carrying sodium citrate were prepared from DOPC, cholesterol and biotin-amidocaproyl-DSPE, Remote loading was used to entrap doxorubicin in liposomes. Streptavidin linker bridged biotinylated microbubbles and liposomes, with pendant complex formation. C57BL/6 mice were injected subcutaneously with MC38 colon carcinoma cells (J. Schlom, NIH); therapy has been initiated as tumors reached 4-5 mm. Doxorubicin-liposome-microbubble complexes were injected intravenously (6 mg/kg doxorubicin). Contrast ultrasound imaging with Sequoia 512 (nondestructive CPS imaging, 7 MHz, MI 0.2) was used to monitor the particles in the tumor vasculature. Therapeutic ultrasound treatment with Birtcher Megason apparatus transducer pointed at the tumor was initiated immediately after complex injection (0.6 W/cm2, 1 MHz continuous sine wave applied for 3 s with 10 s interval between ultrasound pulses to ensure replenishment of drug complexes in the vasculature).

## Results and conclusions

Doxorubicin-liposome-microbubble complexes have been prepared. These pendant particles demonstrated excellent drug load (>1 pg doxorubicin per complex, an order of magnitude higher than what was demonstrated earlier with doxil-like liposomes). Thus, administering 6 mg/kg doxorubicin became feasible, and was performed repeatedly, twice a week for the first two weeks, and then repeated once a week; ultrasound imaging confirmed tumor perfusion with drug carrier. Mice did not lose body mass due to treatment. In response to doxorubicin particle administration combined with insonation, significant suppression of tumor growth was achieved, as compared with control untreated animals (p<0.05); some of treated tumors have reduced size, while untreated controls demonstrated accelerating tumor size increase. Fluorescence microscopy of frozen tumor tissue sections confirmed doxorubicin deposition. In conclusion, repeated administration of doxorubicin-liposome-microbubble particles combined with ultrasound treatment of the tumor under ultrasound imaging guidance achieved significant suppression of tumor growth in a subcutaneous murine tumor model.

